# A Surprising Cause of Liver Abscesses in a Post-Chemotherapy Patient: Herpes Simplex Virus

**DOI:** 10.7759/cureus.17744

**Published:** 2021-09-05

**Authors:** Rukevwe Ehwarieme, Apeksha N Agarwal, Rahaf Alkhateb, Jason E Bowling, Gregory M Anstead

**Affiliations:** 1 Medicine/Infectious Diseases, University of Texas Health, San Antonio, San Antonio, USA; 2 Pathology, University of Texas Health, San Antonio, San Antonio, USA; 3 Medicine/Infectious Diseases, South Texas Veterans Healthcare System, San Antonio, USA

**Keywords:** herpes simplex virus (hsv), hepatitis, hepatic abscess, valacyclovir, hepatic necrosis

## Abstract

Herpes simplex virus (HSV) hepatitis is a rare complication of HSV infection, and a rare cause of hepatitis. It is often fatal, especially if the diagnosis and treatment are delayed. Herein, we describe the case of a 31-year-old female with a history of receiving cytotoxic cancer chemotherapy five months prior who presented with a one-week history of worsening abdominal pain and fever. She was noted to have an outbreak of genital herpes at the time of presentation. Computed tomography (CT) scan of the abdomen showed innumerable hypodensities compatible with hepatic micro-abscesses. A specimen from a subsequent liver biopsy revealed HSV-type cytopathic changes and nuclear staining with an anti-HSV immunohistochemical stain. She was initially started on high-dose oral valacyclovir for genital herpes and was noted to have rapid clinical improvement prior to the histopathologic diagnosis of HSV hepatitis. She achieved full recovery while on oral valacyclovir. This is the first reported case of HSV hepatitis treated with oral valacyclovir and the third reported case of HSV hepatitis mimicking pyogenic abscesses on abdominal imaging. With the high mortality rate associated with HSV hepatitis, one should consider the diagnosis in all patients with multifocal liver lesions of unknown etiology, especially if genital herpes is present at the time of presentation, or in patients who are immunocompromised.

## Introduction

Hepatitis is a rare complication of herpes simplex virus (HSV) infection. HSV hepatitis can sometimes progress to fulminant hepatic failure, and it is often fatal. Untreated HSV hepatitis or a delay in treatment can result in an 80%-90 % mortality rate. Thus, early diagnosis and treatment are imperative to reduce morbidity and mortality [1]. We present the case of a 31-year-old female with a history of chemotherapy for cervical cancer who presented with acute onset of abdominal pain, fever, and genital herpes. A computerized tomography (CT) of the abdomen showed multiple micro-abscesses of the liver. Disseminated HSV infection was diagnosed after both genital and a hepatic biopsy specimen tested positive for the presence of HSV. She was successfully treated with valacyclovir.

## Case presentation

A 31-year-old female presented to the emergency department with abdominal pain and fever of one-week duration. The patient had a history of stage III cervical cancer and had received external beam radiation therapy and chemotherapy with cisplatin four months prior to presentation. She described progressively increasing right upper quadrant pain radiating to her epigastrium. Associated symptoms included nausea, vomiting, chills, and fever of 38.8^o^C. 

On initial presentation, she was toxic-appearing, febrile at 39.5^o^C, with a pulse of 130 beats per minute, and normal blood pressure and oxygen saturation. Her physical examination was notable for right upper quadrant tenderness and guarding, without rebound, rigidity, or palpable masses. She also had multiple vesicles on the external genitalia; she denied any prior history of orolabial or genital herpes. Laboratory data (Table 1) at time of presentation was notable for transaminase and alkaline phosphatase elevation, with aspartate transaminase 1510 IU/L (upper limit of normal (ULN) 31 IU/L), alanine transaminase 733 IU/L (ULN 36 IU/L) and alkaline phosphatase 237 (ULN 117 IU/L). The total bilirubin was normal at 0.6 (ULN 1.2 mg/dL), with an albumin level of 2.0 g/dL (reference range (RR) 3.2-5.0 g/dL) and INR of 1.5 (ULN 1.2). Liver function tests and the creatinine level had been normal three months prior. Her white blood cell count (WBC) at the time of admission was low at 0.95 K/µL (RR 3.40-10.40 K/µL), with absolute neutrophil and lymphocyte counts of 0.45 K/µL (RR 1.50-6.60 K/µL) and 0.05 K/µL (RR 0.9-3.1 K/µL), respectively. The hemoglobin level and platelet count were within the normal ranges. Three months prior, her WBC was 2.38 K/µL, with an absolute neutrophil count of 2.07 K/µL and an absolute lymphocyte count of 0.05 K/µL. Other evaluations, including an acetaminophen level, viral hepatitis panel, *Entamoeba histolytica* IgG, galactomannan, 1,3-beta-D-glucan, *Coccidioides* IgM/IgG, *Histoplasma* urine antigen, rapid plasmin reagin, and serologic tests for cytomegalovirus, Epstein-Barr virus, and the human immunodeficiency virus 1/2 were negative. Urinalysis, urine culture, and blood cultures were also negative. She was started empirically on vancomycin, cefepime, metronidazole, and micafungin. In addition, valacyclovir 1000 mg orally three times a day for genital herpes in an immunocompromised host was started. She was evaluated by the hematology service and had an extensive evaluation for the cause of her neutropenia. Her peripheral smear showed no evidence of myelodysplasia, leukoerythroblastosis, or atypical lymphocytosis. Evaluation for hemolysis and iron and vitamin deficiencies was unrevealing. She received a single treatment with filgrastim, with a subsequent improvement in her neutrophil count.

**Table 1:** Summary of patient’s laboratory values. AST: aspartate aminotransferase; ALT: alanine aminotransferase; ALK-P: alkaline phosphatase; T-bili: total bilirubin; ANC: absolute neutrophil count; ALC: absolute lymphocyte count.

Hospital day/event	ALT (U/L)	AST (U/L)	ALK-P (U/L)	T-Bili (mg/dL)	WBC (K/µL)	ANC (K/µL)	ALC (K/µL)
1/Filgrastim started	733	1510	329	0.5	0.59	0.48	0.05
2/Valacyclovir started	865	1956	413	0.6	1.29	1.09	0.04
3	568	920	265	0.4	2.20	1.92	0.04
7	149	67	108	0.5	9.95	7.48	1.05
9	99	39	204	0.4	10.55	8.28	1.25

**Figure 1:**
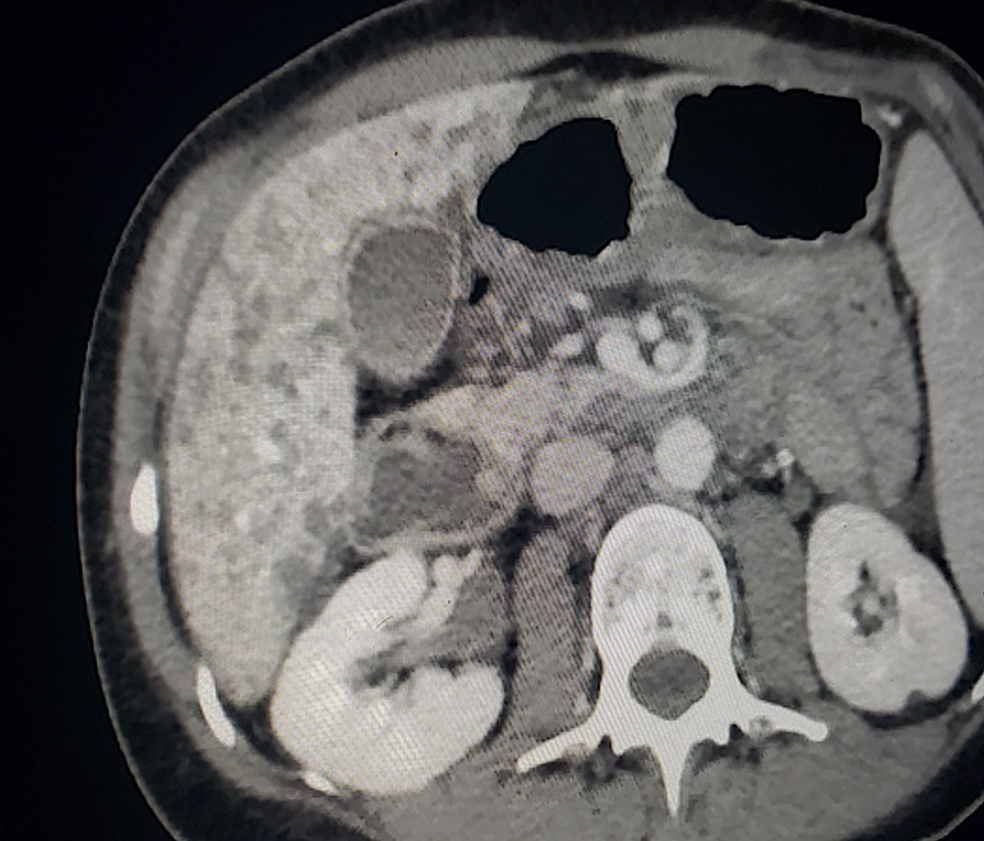
Computerized tomography of abdomen and pelvis with IV contrast. There were innumerable small hypodensities throughout the liver, new from a prior PET CT imaging five months prior. These were concerning for microabscesses from bacterial or fungal infection. Nonspecific adjacent inflammatory changes were also apparent.

**Figure 2:**
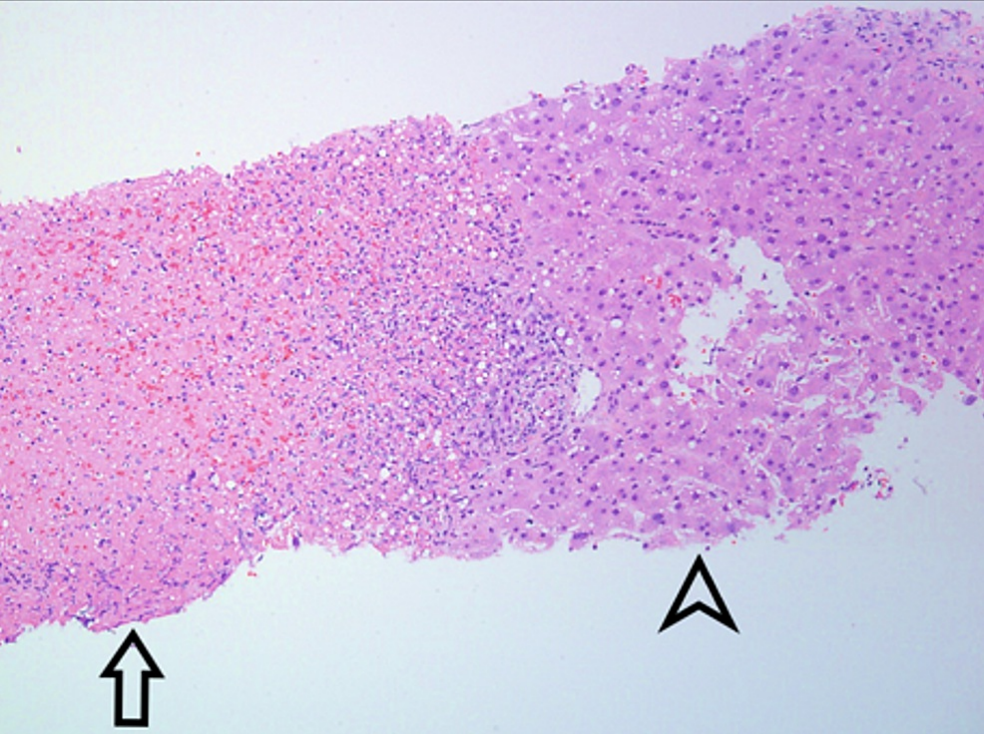
Photomicrograph of liver biopsy showing a zone of necrosis (left half of image, indicated by arrow) and a zone of viable hepatocytes (right half of image, indicated by arrowhead). Magnification 100X (hematoxylin and eosin (H&E)).

**Figure 3:**
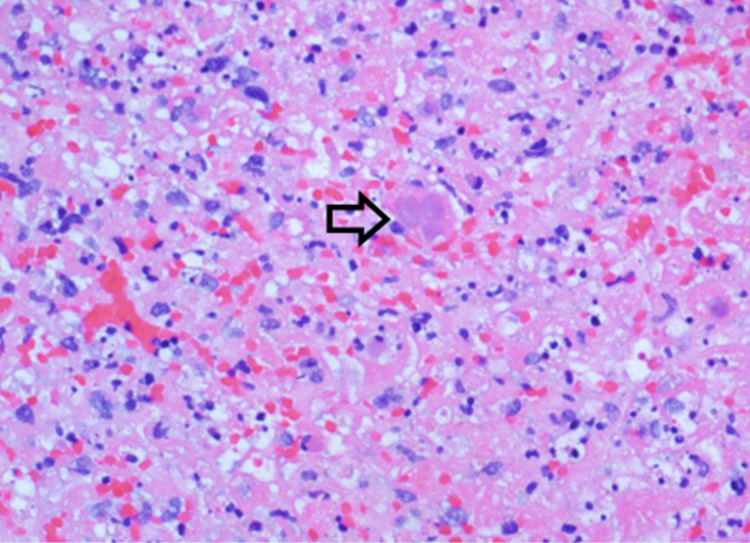
Photomicrograph of liver biopsy section showing a degenerating hepatocyte with smudged chromatin and multinucleation consistent with viral cytopathic changes (arrow) in a background of necrosis and lymphocytic infiltrate. Magnification 400X (hematoxylin and eosin (H&E)).

A CT scan of the abdomen and pelvis with contrast demonstrated innumerable small hypodensities throughout the liver, which were new compared to a positron emission tomography (PET) scan performed five months prior to admission. These findings were concerning for micro-abscesses from a bacterial or fungal infection (Figure 1). The patient subsequently underwent a CT-guided core needle biopsy of the liver. Histopathologic exam of the liver biopsy specimen showed hepatocytes with inflammation and multifocal confluent necrosis (Figures 2, 3). Within the area of necrosis, there were degenerating hepatocytes with smudged chromatin and multinucleation consistent with a viral cytopathic effect. Additional specimen testing, including bacterial culture, Grocott methenamine silver (GMS) stain for fungal organisms, Fite stain for mycobacteria, immunohistochemical stain for cytomegalovirus, and Epstein-Barr virus encoding region (EBER) in situ hybridization, were all negative.

**Figure 4:**
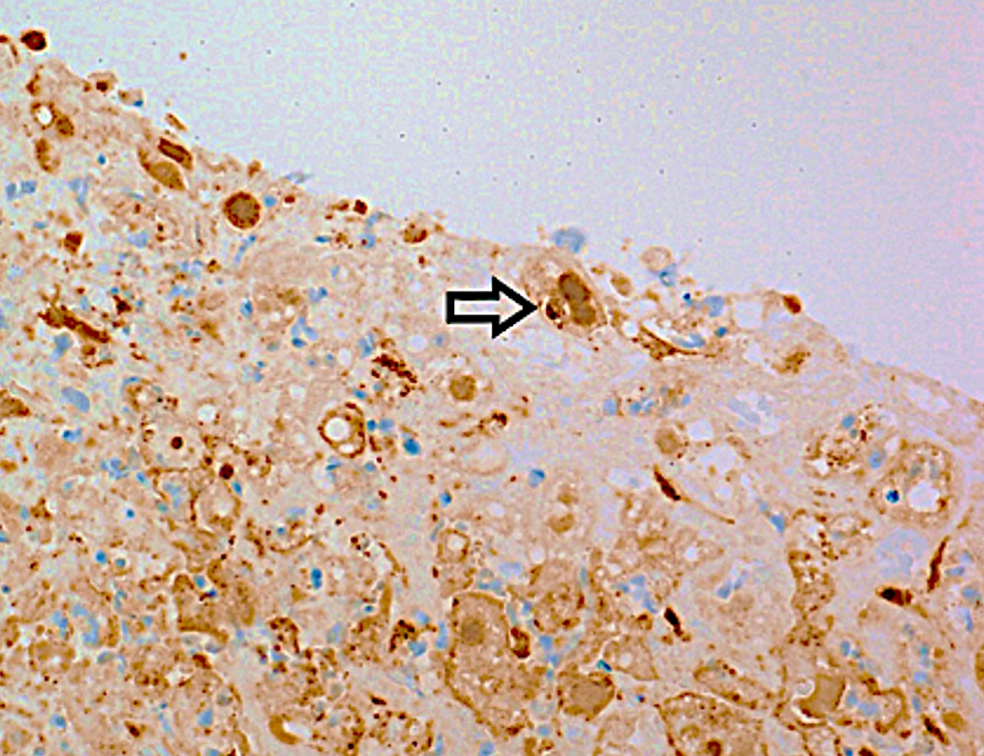
Photomicrograph of liver biopsy section with immunohistochemical staining for HSV-1/2 demonstrating positive nuclear staining in a multinucleated hepatocyte (arrow), magnification 200X. This stain does not differentiate HSV-1 from HSV-2.

Immunohistochemical staining was positive for HSV (which did not distinguish HSV-1 and HSV-2) (Figure 4). Serologic titers for combined HSV-1 and -2 IgM and IgG were 5.0 and 2.85, respectively (RR for both IgM and IgG, 0.89 or less = not detected). A swab of the genital vesicular lesions tested positive for HSV-2 by the polymerase chain reaction (PCR).

**Figure 5:**
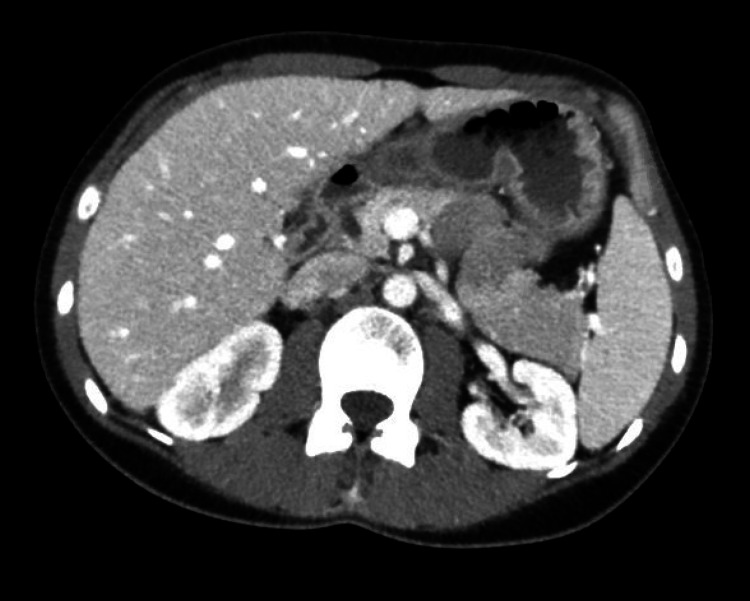
Computerized tomography of abdomen and pelvis with IV contrast, six months after completion of a course of high-dose valacyclovir. The liver was normal in size and morphology, with interval resolution of previously seen diffuse nodular hypoattenuating lesions.

Based on the immunohistochemical findings, she was diagnosed with HSV hepatitis, likely due to HSV-2 based on the PCR result from the concurrent genital lesions. Forty-eight hours after the initiation of valacyclovir, her fevers resolved, and her transaminase levels began to normalize. The antibiotics were discontinued and valacyclovir was continued at the same dose for three weeks and was then transitioned to once-daily dosing. At an outpatient appointment four weeks after discharge, she was asymptomatic with normal liver function tests and a normal WBC and differential counts. The patient was lost to follow-up for seven months but did have a repeat CT scan of her liver seven months after her hospitalization; the liver had a completely normal radiographic appearance at that time (Figure 5).

## Discussion

HSV hepatitis is reported to cause less than 0.8% of all cases of acute liver failure, and less than 2% of viral causes of acute liver failure [2]. Most cases have been reported in immunosuppressed patients and women in their third trimester of pregnancy, although up to 25% of cases occur in immunocompetent individuals [3]. Due to a lack of awareness and its nonspecific clinical presentation, the diagnosis of HSV hepatitis is frequently made post-mortem [3].

Hepatitis due to HSV infection can be due to either HSV-1 or HSV-2 [4]. Common clinical features of HSV hepatitis include fever, elevated aminotransferase levels (often >1000 U/L), leukopenia, encephalopathy, coagulopathy, and acute renal failure. About 90% of HSV hepatitis patients have a liver profile termed “anicteric hepatitis,” which refers to a 100- to 1000-fold increase in transaminase levels (AST greater than ALT) and a relatively normal or low bilirubin level [5]. Older age, male sex, immunocompromised status, the degree of aminotransferase elevation, and the presence of coagulopathy and encephalopathy have been significantly associated with progression to death or the need for liver transplantation [3]. In the case of our patient, she had a history of squamous cell carcinoma of the cervix and had received chemotherapy and radiation treatment three months prior to her admission, with neutropenia and lymphocytopenia on admission, one could postulate that the leukopenia is related to disseminated HSV as this has been reported in 43% of other cases [4].

Although the pathogenesis of HSV hepatitis is not well established, potential mechanisms of acute liver failure include: (i) a large HSV inoculum overwhelming the immune system; (ii) dissemination of mucosal herpetic lesions in the setting of a suppressed immune system; (iii) acute HSV infection, superimposed on a latent HSV reactivation; or (iv) re-infection with a proposed strain of hepatovirulent HSV [6].

Liver biopsy is the gold standard diagnostic test for HSV hepatitis [7]. In a patient in whom a biopsy is contraindicated due to coagulopathy, HSV serum PCR is also a highly sensitive and specific test, allowing for rapid diagnosis and early initiation of therapy [8]. On the other hand, serologic testing has a low sensitivity and specificity as it can take two to three weeks for serological conversion after HSV exposure. The HSV IgM antibody titers typically increase four-fold about two to four weeks after the infection [8]. In the case of our patient, her positive PCR result was available in three days and biopsy result in five days. Her serologic testing for HSV-1 and -2 IgM and IgG was also positive. While many disseminated HSV infections occur with primary infection, these results suggest that the HSV hepatitis observed in this patient was secondary to reactivation of genital HSV infection in the setting of a suppressed immune system after her chemotherapy treatment, or an acute on chronic infection with a hepatovirulent strain. Imaging findings of HSV hepatitis are non-specific and are usually findings of hypodense lesions with associated hepatomegaly [9], in our patient her abdominal CT scan showed liver micro-abscesses. Although the patient’s radiographic findings were concerning for a pyogenic process, typically pyogenic abscesses present with leukocytosis in 89% of cases and the ALT elevation is much less (180.3 ± 149.9 U/L in one series) than in herpes hepatitis [10]. Interestingly, this is only the third reported case of HSV hepatitis presenting with CT imaging findings mimicking an abscess (see Table 2) [11,12]. Case 1 of Table 2 occurred in an immunocompetent patient and Case 2 in an immunocompromised one; in both cases, the diagnosis was made by liver biopsy with immunohistochemical staining. The immunocompromised patient of Case 2 succumbed to the infection, despite acyclovir treatment.

**Table 2:** Previous cases of herpes simplex hepatitis presenting as hepatic microabscesses.

No/ [ref]	Age (years), sex	Brief course
1/[11]	67, male	History of prostate cancer 7 years post-prostatectomy with no residual disease. Presented with epigastric pain, fatigue, and elevated serum transaminase levels. CT and MRI scans revealed multiple liver abscesses. Serologic testing showed elevated HSV-1 and HSV-2 antibody levels. Liver biopsy with immunostaining for HSV-2 was positive, and pathologic analysis revealed HSV-type cytopathic changes. He was treated with a 4-week course of IV acyclovir and achieved full recovery. Follow-up CT scan of abdomen and pelvis showed persistence of hepatic nodules.
2/[12]	23, female	History of Crohn’s disease on prolonged corticosteroids. Hospitalized for fever, diarrhea, and elevated serum transaminase levels. CT scan revealed intramural sigmoid and multi-lobar liver abscesses. Sigmoid resection and liver biopsy were performed at laparotomy. Histologic examination of the liver and colon with immunostaining for HSV-2 were positive, and pathologic exam revealed HSV-type cytopathic changes in both the sigmoid colon and liver specimens. The patient subsequently died, despite parenteral acyclovir.

Despite its high mortality, HSV hepatitis is one of the few treatable causes of acute liver failure. Early initiation of acyclovir has been shown to decrease morbidity and mortality [3]. Previous cases have shown that early initiation of acyclovir (within three days of presentation) leads to better outcomes due to the cessation of viral replication and dissemination of infection. While there have been no controlled studies evaluating the treatment of HSV hepatitis, high dose intravenous acyclovir, 10 mg/kg, for up to 21 days is generally recommended [3]. Interestingly, our patient’s case is the first reported with clinical resolution using oral treatment with valacyclovir alone. While the valacyclovir was initiated as treatment for genital herpes, our patient was noted to have had marked clinical improvement 24 to 48 hours following the initiation of therapy. She was treated with 1000 mg oral valacyclovir three times a day for two weeks, and then as suppression therapy of valacyclovir 1000 mg daily.

## Conclusions

In conclusion, HSV hepatitis is a potentially lethal infection that is often unrecognized. When suspected, early initiation of treatment with acyclovir, especially within three days of presentation, can have significant morbidity and mortality benefit. In this case, the initial radiographic presentation of hepatic micro-abscesses confused the clinical picture. However, the simultaneous presentation of genital herpes led to the rapid initiation of treatment and rapid improvement of hepatic dysfunction. Although not the case in our patient, HSV hepatitis should also be suspected in immunocompromised patients who present with transaminase elevation without apparent genital lesions. Though diagnosis with biopsy is the gold standard, HSV PCR could serve as a substitute marker and aid in rapidly diagnosing the disease. Imaging findings in HSV hepatitis are non-specific and it rarely presents as multi-focal liver abscesses.
